# Effectiveness of intervention strategies exclusively targeting reductions in children’s sedentary time: a systematic review of the literature

**DOI:** 10.1186/s12966-016-0387-5

**Published:** 2016-06-09

**Authors:** Teatske M. Altenburg, Joana Kist-van Holthe, Mai J. M. Chinapaw

**Affiliations:** Department of Public and Occupational Health, VU University Medical Center, EMGO Institute for Health and Care Research, Van der Boechorststraat 7, 1081 BT Amsterdam, The Netherlands

**Keywords:** Prevention, Paediatric, Screen time, Television, Sitting

## Abstract

An increasing number of interventions targeting sedentary behaviour in children have emerged in recent years. Recently published reviews included sedentary behaviour and physical activity interventions. This review critically summarizes evidence on the effectiveness of intervention strategies that exclusively targeted reducing sedentary time in children and adolescents. We performed a systematic literature search in Pubmed, Embase and the Cochrane Library through November 2015. Two independent reviewers selected eligible studies, extracted relevant data and rated the methodological quality using the assessment tool for quantitative studies. We included 21 intervention studies, of which 8 studies scored moderate on methodological quality and 13 studies scored weak. Four out of eight moderate quality studies reported significant beneficial intervention effects.

Although descriptions of intervention strategies were not always clearly reported, we identified encouragement of a TV turnoff week and implementing standing desks in classrooms as promising strategies. Due to a lack of high quality studies and inconsistent findings, we found no convincing evidence for the effectiveness of existing interventions targeting solely sedentary behaviour. We recommend that future studies apply mediation analyses to explore which strategies are most effective. Furthermore, to increase the effectiveness of interventions, knowledge of children’s motives to engage in sedentary behavior is required, as well as their opinion on potentially effective intervention strategies.

**Electronic supplementary material:**

The online version of this article (doi:10.1186/s12966-016-0387-5) contains supplementary material, which is available to authorized users.

## Background

Children nowadays spend a large amount of their time in sedentary behaviours with average values of up to nine hours per day for total sedentary time and up to four hours per day for screen time [1–5]. Importantly, many children exceed the World Health Organization (WHO) recommendation of limiting electronic media use (i.e. <1 h/day for 2–4 year olds and <2 h/day for 5–17 year olds). For example, up to 80 % of 12–17 year olds spend more than 2 h/day on screen behaviours [4, 6].

To date, evidence regarding the relationship between sedentary behaviour and health indicators in children is not convincing [7–9], partly due to a lack of methodologically sound studies [10]. Nevertheless, the growing public health concern regarding the health effects of excessive sedentary behaviour in children has led to an emerging number of interventions targeting sedentary behaviour in children and adolescents in recent years [11–14].

A number of reviews have been published on the effectiveness of interventions targeting sedentary behaviour [15–25], as well as a review of reviews [26]. However, a number of studies included in these reviews included strategies targeting promotion of moderate-to-vigorous physical activity. It is therefore not known which intervention strategies resulted in sedentary behaviour change, and which intervention strategies that exclusively target reductions in sedentary behaviour are effective.

Therefore, the aim of the present review was to critically summarize the evidence regarding the effectiveness of interventions that exclusively targeted reductions in sedentary time in children and adolescents (0 to 18 years old), without strategies promoting physical activity simultaneously. Additionally, we aimed to identify effective intervention strategies to reduce sedentary time. Though current guidelines focus on screen-based sedentary behaviour, recent evidence highlights the importance of limiting prolonged sitting [27–29]. Therefore, we did not limit our review to interventions targeting a specific type of sedentary behaviour.

## Review

### Methods

#### Literature search

We performed a systematic literature search in Pubmed, Embase and the Cochrane Library from inception through November 2015. The search strategy included terms related to ‘interventions’ (e.g. randomized controlled trial, controlled trial, control group) in AND-combination with terms related to sedentary behaviour (e.g. screen time, television, computer use, sitting). The search was limited to studies in children and adolescents (i.e. participants aged 0–18 years). Additional file 1 provides the full search. In addition, reference lists were screened for potential additional studies.

#### Inclusion and exclusion criteria

Intervention studies were included if they (i) evaluated interventions (or intervention arms) targeting sedentary behaviour (e.g. TV viewing, computer use, reading, playing board games etc.) in children and adolescents (aged 0 to 18 years old), and (ii) included time spent in sedentary behaviour as an outcome measure. Studies were excluded if they also included strategies that promoted increases in physical activity. We included only full-text studies that were published in the English language in peer-reviewed journals.

#### Selection process and data extraction

Two reviewers (TA and JK) independently checked all identified titles and abstracts to establish potential relevant studies. Next, two reviewers (TA and JK) independently screened the full-text papers to check if they met the inclusion criteria. Any discrepancies were resolved through discussion and, if consensus was not reached, with a third reviewer (MC).

The following data were extracted from all included studies, using a structured form: (i) participant characteristics; (ii) intervention strategies; (iii) intervention setting; (iv) intervention duration and follow-up; (v) description of control group (vi) sedentary behaviour outcome; and (vii) results of the study.

#### Methodological quality assessment and data synthesis

Two reviewers (TA and MC) independently rated the methodological quality of all included studies using the quality assessment tool for quantitative studies of the Effective Public Health Practice Project (EPHPP) [30]. This tool uses three response options (strong, moderate, weak) on the following eight quality criteria: selection bias, study design, confounders, blinding, data collection methods, withdrawals and dropouts, intervention integrity, and analysis (see Additional file 2). Any discrepancies were resolved through discussion. The methodological quality of a study was rated as strong when at most one of the quality criteria was scored as weak and two as moderate. A study was rated as moderate when at most two weak quality criteria were scored as weak. When more than two quality criteria were scored as weak, a study was rated as weak.

To synthesize the study results, we applied a best-evidence synthesis [31], in accordance with the Cochrane Collaboration. Using this method, the number of studies, their methodological quality and the consistency of the results are taken into account:

- Strong evidence – consistent findings in ≥ 2 studies of strong quality;
- Moderate evidence – consistent findings in ≥2 studies of moderate quality;
- Conflicting/insufficient evidence – conflicting findings or lack of moderate/high quality studies.

### Results

**Figure 1:**
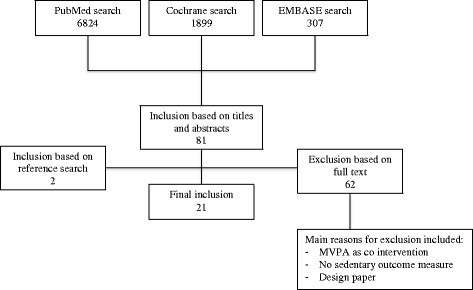
Flowchart of included papers

The literature search yielded a total of 9825 hits: 7348 in Pubmed, 532 in Embase and 1945 in Cochrane. After removing duplicates and checking eligibility, we included 19 relevant papers. Two additional papers were included after screening reference lists, resulting in a total of 21 included papers. Figure 1 summarizes the flowchart of included papers.

#### Participant, study and intervention characteristics

Table 1 presents the participant and intervention characteristics of included studies, sorted by age range. Sample sizes ranged from 11 to 1569 (20 to 100 % boys) for the intervention group and 7 to 1578 (18 to 100 % boys) for the control group. Mean age of the participating children ranged from 3.1 to 11.3 years old. Seven studies included children aged 2.5 to 7 years [11, 13, 32–36] and 14 studies included children aged 7 to 12 years [12, 14, 37–48].

**Table 1:** Participant and intervention characteristics – sorted by age range

		Intervention characteristics				Control
Ref	Participants	Intervention strategies	Setting	Duration	Follow up	Description
Children aged 2.5 – 7 years						
Birken et al. [11]	Intervention *n* = 64; 44 %M; 3.1 ± 0.2 yrs Control *n* = 68; 49 %M; 3.1 ± 0.1 yrs	*General description:* Parents engaged in a 10-min counselling on the health impact of screen time in children and strategies to reduce screen time (e.g. removing TV from children’s bedroom, budgeting of children’s screen time) and on safe media use. *Theory-based*: Social cognitive theory *Knowledge transfer:* Parents received standard behavioural counselling on safe media use and three Canadian Pediatric Society hand-outs. *TV turnoff*: One week encouraged, children received rewarding for days without TV. *Parental skills:* Parents rewarded children for days without TV. *Child involvement*: Children provided parents a story about TV viewing and created a list of non TV-related activities (contingency planning).	Family/home	1-year	No	Parents received standard counselling on safe media use and a Canadian Pediatric Society hand-out*.*
Dennison et al. ^b^ [32]	Intervention *n* = 43; 20 %M; 3.9 ± 0.1 yrs Control *n* = 34; 18 %M; 4.0 ± 0.1 yrs	*General description:* Preschool/daycare staff engaged in seven 20-min sessions on reducing children’s TV viewing, including encouraging parents/staff to read stories to children daily, family mealtime with TV turned off, a party was held for children and staff for surviving a week without TV and a booster session during the National TV-Turnoff week. Children were rewarded for ‘the best reader at home’ and for days not watching TV. *Knowledge transfer:* Parents received take-home materials of all sessions and a brochure (by the American Academy of Pediatrics). *TV turnoff*: One week encouraged. *Parental skills:* Parents were asked to keep a diary to increase awareness of their child’s TV viewing and to reward children for each day without TV viewing. *Child involvement*: Children discussed alternative activities to watching TV, made ‘no TV’ signs, planned a party for a week without TV.	Preschool/daycare; family/home	39 weeks	No	Usual curriculum; materials and ideas for activities about health and safety were provided tot day care or preschool staff and information and materials for at-home activities were mailed to parents. Eight monthly sessions, each with a different health or safety topic, were provided for the 2nd school year.
Epstein et al.^a^ [33]	Intervention *n* = 34; 53 %M; 5.8 ± 1.2 yrs Control *n* = 36; 53 %M; 6.1 ± 1.3 yrs Children with a BMI > 75^th^ percentile for age and sex	*General description:* Weekly time budgets for TV viewing, computer use and associated behaviours were set during home visits, using a TV control device. Families received ideas for alternatives to sedentary behaviour, a tailored monthly newsletter with parenting tips to reduce sedentary behaviour, and information about how to rearrange the home environment to reduce access to sedentary behaviour. *Knowledge transfer*: Parents received monthly newsletters including tips to reduce SB and how to arrange the home environment to reduce access to SB. *TV control device:* Families received a TV allowance, attached to each TV and each computer monitor in the home. *Parental skills:* Parents were instructed to praise their children for reducing TV viewing and engaging in alternate behaviours. *Goal setting*: Research staff set weekly TV budgets, which were weekly reduced by 10 % to a maximum of 50 %, based on baseline amounts. When the budget was reached, the TV or computer could not be turned on for the remainder of the week. The research staff rewarded children for amount of time under budget.	University children’s hospital; family/home	2 years (measures every 6 months from baseline)	No	Free access to TV and computers and 2-dollar budget per week for participating. Families received a newsletter providing parenting tips, sample praise statements, and child-appropriate activities and recipes.
Haines et al.^b^ [13]	Intervention *n* = 55; 56 %M; 4.1 ± 1.1 yrs Control *n* = 56; 48 %M; 4 ± 1.1 yrs Children from low-income and racial/ethnic minority families	*General description:* Parents engaged in individually tailored counselling (motivational coaching by health educator) to encourage behavioural change (four 60-min home visits and four 20-min telephone calls). Educational materials and incentives, and weekly text messages on adoption of household routines were mailed (twice weekly for 16 weeks and weekly for the last 8 weeks). Home visits targeted behavioural change: review progress and setbacks, goals and tools. Phone calls targeted parents’ progress on making change, provide support and reinforce study messages. Focus on limiting TV time, eating more meals together as a family with TV off and removing the TV from the child's bedroom. *Theory-based*: Social ecological model. *Knowledge transfer:* Parents engaged in counselling sessions, and received educational materials and text messages. *TV control device:* In subsample of 30 participants (±25 %) *Parental skills:* Parents were coached on goal setting for their child’s behaviour and provided with tools to support their child’s behaviour change. Parents with TV control device were assisted with goal setting to reduce total viewing time. *Goal setting*: Parents encouraged to set goals for their child’s behaviour.	Family/home	6 months	No	Control group received four monthly mailed packages including educational materials on reaching developmental milestones during early childhood and low-cost incentives (e.g. colouring books).
Taveras et al. [34]	Intervention *n* = 253; 52 %M; 4.8 ± 1.2 yrs Control *n* = 192; 51 %M; 5.2 ± 1.1 yrs Children with a BMI between 75^th^ and 85^th^ percentile	*General description:* Pediatric nurse practitioners conducted four 25-minute in-person chronic disease management visits and three 15-minute phone calls (motivational interviewing) in the first year of the intervention. Posters in the waiting room highlighting the targeted behaviours: less than 1 hour per day TV/video viewing, removing TV from or avoiding putting a TV in the child’s bedroom. For the chronic disease managements visits, educational modules were developed targeting TV viewing and matching the family’s stage of readiness to change. Small incentives were provided to further support behavioural change. *Theory-based*: Chronic care model. *Knowledge transfer*: Families received education on TV viewing, matched with their stage of readiness to change, and were provided with tools for self-management support, an interactive website with educational materials. *TV control device:* Electronic monitoring device offered to families to assist with the goal of reducing TV viewing.	Clinic; family/home	1 year (mid-intervention results)	No	Usual care including well-child care visits and follow-up appointments for weight checks
Yilmaz et al. [36]	Intervention: *n* = 176; 65 %M; 3.5 ± 1.2 yrs Control: *n* = 187; 66 %M; 3.5 ± 1.3 yrs	*General description:* Families were exposed to four intervention components at two week intervals: 1) printed materials and interactive CD’s; 2) counselling call; 3) age-appropriate picture book showing a role-model families, and including knowledge on screen time; and 4) stories of role-model families. *Theory-based:* Social cognitive theory. *Knowledge transfer:* Parents received several materials and counselling calls. *Parental skills:* Parents received encouraging counselling call.	Family/home	2 months	2, 6 and 9 months	-
Zimmerman et al. [35]	Overall 2.5–4.5 yrs Intervention *n* = 34; %M not reported Control *n* = 33; %M not reported	*General description:* Families received written materials and four monthly newsletters targeting 1) to reduce the child’s media viewing to 1 h per day or less and 2) to replace commercial media viewing with educational viewing. A case manager contacted (phone or email) families to facilitate behaviour change, with communication in four domains: 1) positive/negative effects of TV on child’s health and development, 2) encouragement to the mother in building confidence to modify a child's TV viewing, 3) strategies for modifying the child’s TV viewing and 4) assessment and counselling in the parent’s stage of change for modifying TV viewing. *Knowledge transfer*: Parents received written materials and four monthly newsletters, and were contacted by case manager. *Parental skills*: Parents were provided with encouragement in building confidence to modify the child’s TV viewing and strategies for modifying the child’s TV viewing. *Goal setting*: Parents were encouraged by the research staff to reduce their child’s media viewing to 1 hour per day or less, and replace commercial TV viewing with educational viewing.	Family/home	4 months	No	Injury-prevention and pre-schooler safety targeted. Parents were asked to promote their child’s safety in several areas, for example regular use of bike helmets, regular and appropriate use of car seats, home fire safety.
Children aged 7 – 12 years						
Cardon et al. [44]	Intervention *n* = 19; 8.3 ± 0.6 yrs; 53 %M Control *n* = 23; 8.1 ± 0.5 yrs; 48 %M	*General description*: Encouragement of movement in the school, by 1) work organisation encouraging movement (e.g. information stations); 2) circumstances creating movement (e.g. stand-at places of work); and 3) behavioural influences (e.g. good examples). Ergonomic furniture in classroom allowing varying working postures and contributing to dynamic sitting. All tables have inclinable tops (minimum inclination of 16°), more floor space available in the classroom for variation in daily working routines (e.g. corner for reclining, mats on the floor). *Knowledge transfer*: Children provided with good examples, encouragement and training on awareness of healthy behaviour. Also knowledge on posture-physiology and motivation for lasting behaviour change. *Change in environment*: Standing work places and work organisation to encourage movement/reduce sitting.	School	1.5 years	No	Traditional furniture
Carson et al.^a^ [12]	Intervention *n* = 74; 37 %M; 7.9 ± 1.4 yrs Control *n* = 64; 59 %M; 8.1 ± 0.4 yrs	*General description:* Children received class-learning messages (9 out of 18 by mid-intervention), one standing class lesson each day (±30 minutes) and a 2-min light intensity activity break every 30 minutes within each 2-hour teaching block (teachers were provided with timers). Standing easels were placed in class so that could rotate learning activities at standing desks. Children completed homework tasks on reducing sitting time (alone and with parents). *Theory-based*: Social cognitive theory, behavioural choice theory and ecological systems theory *Knowledge transfer:* Children received key-learning messages in class on raising awareness, self-monitoring, goal setting, behavioural contracts, social support and feedback and reinforcement; parents received nine newsletters including these key-learning messages, family based activities to complete with their child and information on how to reduce their child’s screen time (e.g. effective use of rules). *Change in environment*: Standing easels in class to alternate sitting with standing.	School; family/home	24 months (mid-intervention results)	No	Usual practice
Epstein et al. ^b^ [38]	Low dose SB *n* = 20; 25 %M; 10.7 ± 1.0 yrs High dose SB *n* = 20; 40 %M; 10.6 ± 1.1 yrs	*General description:* Families engaged in 16 weekly meetings, followed by 2 biweekly and 2 monthly meetings on healthy diet and decreasing SB, with separate groups for children and parents. Children were reinforced for reducing SBs that compete with being active or set the occasion for eating (viewing TV/videotapes, playing computer games, talking on the phone, or playing board games). Academically relevant SBs were not targeted. *Knowledge transfer:* Families received parent and child workbooks on weight control, self-monitoring, the traffic light diet, specific activity program, behaviour change techniques and maintenance of behaviour change. *Parental skills:* Parents rewarded children when meeting goals. *Goal setting:* Parents and children set goals and reinforcers to be provided when meeting the goal.	Childhood obesity research clinic; family/home	6 months	12 and 24 months	No control group. Low dose or high dose groups for reducing SB.
Epstein et al. ^b^ [37]	Intervention *n* = 32; 34 %M; 9.8 ± 1.4 yrs Control *n* = 30; 40 %M; 9.9 ± 1.2 yrs Children between 20 % and 100 % overweight	*General description:* Families engaged in 16 weekly meetings, two biweekly meetings, 2 monthly meetings on reducing SB to no more than 15 hours per week, using shaping steps of 25, 20 and 15. Topics were self-monitoring, behavioural change and maintenance of change. Children were awarded for meeting their goals (based on baseline values) – i.e. reinforcement group. Families recorded targeted sedentary behaviour times in habit books. *Knowledge transfer*: Families engaged in meetings and received habit books. *Parental skills:* Parents were instruct to monitor their child’s SB using habit books, and were taught to review habit books daily with their child, and praise and reward their child for meeting goals (contract reinforcement system). *Goal setting*: Children were encouraged by the treatment staff to reduce SB to no more than 15 hours per week.	Clinic; family/home	6 months	12 months	Instructions to reduce SB to 15 or fewer per week, change environment to prevent engagement in targeted SB, establish rules regarding SB, and aid sedentary behaviour change (e.g. posting signs indicating sedentary limit and unplugging targeted SB (TVs/PCs)). Positive reinforcement for recording SB (but not for behavioural change) – i.e. stimulus control group.
Escobar-Chaves et al. [39]	Overall *n* = 199; 49 %M; 8.2 ± 0.8 yrs	*General description:* Families engaged in one 2-hour workshop on how to incorporate the five behavioural objectives into their daily routines (including interactive discussion about TV facts and concurrent parent–child activities) and six bimonthly newsletters focusing on five behavioural objectives/steps to reduce media consumption (e.g. TV viewing): 1) reduce TV viewing; 2) turn of TV when nobody is watching; 3) no TV with meals; 4) no TV in the child’s bedroom; 5) engage in fun, non-media related activities. Parents and children worked together on a Fun Family Plan. *Theory-based*: Social cognitive theory. *Knowledge transfer:* Parents engaged in a 2-hour workshop and received six monthly newsletters. *Parental skills*: Parents learned communication skills via role playing (from workshop) and positive peer role model stories (from newsletters). *Child involvement*: Children discussed lessons learned, made a hand puppet as a cue to action and brainstormed about activities besides media consumption, and discussed these with parents.	Family/home	6 months	No	-
Ford et al. [40]	Intervention *n* = 15; 47 %M; 9.5 ± 1.4 yrs Control *n* = 13; 46 %M; 9.6 ± 1.7 yrs	*General description:* Families engaged in a brief standard counselling intervention (5–10 minutes), including discussion of potential problems associated with excessive media use and three brochures from the American Academy of Pediatrics, and engaged in a 15–20 minutes discussion about setting TV budgets. *Theory-based*: Social cognitive theory. *TV control device:* Families received a TV allowance, to monitor and budget TV, videotape and video game use. *Knowledge transfer:* Parents received counselling and a brochure*.* *Parental skills:* Parents engaged in discussion on setting TV viewing budgets (counselling session) and received instructions for monitoring their child’s TV viewing, setting a weekly media budget and helping their child to stick to this budget. *Goal setting*: Parents instructed to set weekly media budgets.	Family/home	4 weeks	No	Standard counselling intervention (5–10 minutes), including discussion of potential problems associated with excessive media use and three brochures from the American Academy of Pediatrics.
French et al. [45]	*n* = 40; 50 %M; 9.0 ± 2.2 yrs	*General description:* TV control devices (attached to every working TV in the home) implemented during initial home visit, followed by five monthly telephone calls. Number of hours programmed on the devices (lower than baseline TV viewing time; recommendation: <2 hrs/day) was discussed and agreed on (by parent) during the home visit. For phones and small screens parents were encouraged to limit their child’s use. Telephone contact, using motivational interviewing, to help parents set goals and make changes in home environment. Additionally, non-caloric beverages were delivered. *TV control device:* 6 months. *Parental skills:* Parents encouraged to set goals for programming of TV viewing time, limit use of phones and small screens and make changes in home environment regarding screens. *Goal setting:* Parents programmed the number of hours of TV viewing.	Family/home	6 months	No	-
Hinckson et al. [46]	Intervention *n* = 23; 9 ± 1 yrs Control *n* = 7; 10 ± 0 yrs	*General description:* Child-adjusted standing workstations were introduced in the classrooms. Exercise balls, beanbags, and mats were available for children to sit when tired. Traditional desks and chairs were removed. *Change in environment*: Standing workstations in class.	School	4 weeks	No	Classrooms with traditional desks and chairs.
Maddison et al. [47]	Intervention: *n* = 127; 57 %M; 11.2 yrs Control: *n* = 124; 56 %M; 11.3 yrs Overweight and obese children	*General description:* Parents were encouraged to change the home environment to facilitate behaviour change of the child and to implement behaviour change strategies (SWITCH). Three elements offered to families: 1) provision of behavioural change strategies by offering education and support; 2) assistance to budget media time, by a TV control device; and 3) an activity pack for children, including options for non-screen based activities. *Theory-based:* Social cognitive theory, behavioural economics theory. *Knowledge transfer:* Parents received cultural relevant education and support to implement strategies in the home environment, and they received newsletters. *TV control device:* 20 weeks *Parental skills:* Parents were encouraged (during a face-to-face meeting) to include praise, positive reinforcements, environmental control budgeting and self-monitoring, positive role modelling.	Family/home	20 weeks	24 weeks	Families continued with their usual behavior and had access to generic SWITCH public website.
Ni Mhurchu et al. [42]	Intervention *n* = 15; 67 %M; 10.4 ± 0.9 yrs Control *n* = 14; 57 %M; 10.4 ± 0.9 yrs	*General description:* Children were encouraged to restrict TV viewing to 1 h per day or less. Parents engaged in a discussion on how to use the TV control device within their household and discussed ideas to manage TV viewing. *TV control device:* Families received an electronic TV time monitor (up to 2 per household). Parents were given 30 tokens, each allowing 30 minutes of TV time. *Knowledge transfer:* Parents were engaged in discussion. *Parental skills*: Parents discussed the usage of the TV control device (e.g. creating rules around household TV viewing) and had the option of blocking out certain time periods to help control the content of TV programmes viewed by children. *Goal setting:* Parents were encouraged by the research staff to restrict their child’s TV viewing to 1 h per day or less.	Family/home	6 weeks	No	Families received verbal advise on general strategies to decrease TV watching (single session).
Robinson et al. [43]	Intervention *n* = 92; 55 %M; 8.9 ± 0.64 yrs Control *n* = 100; 51 %M; 8.9 ± 0.7 yrs	*General description:* Children received 18 lessons of 30–50 minutes, including self-monitoring and self-reporting of TV/video and video game use. A TV turnoff period was encouraged as well as a 7-hour per week budget of TV/video and video game use. Parents received newsletters motivating them to help their children to stay within their budget and suggesting strategies for limiting TV/video and video game use were provided by newsletters. *Theory-based*: Social cognitive theory. *TV turnoff*: Ten days encouraged. *TV control device:* Families received a TV allowance for each TV in the home to help with budgeting. *Knowledge transfer:* Children engaged in lessons on self-monitoring and self-reporting of TV/video and video game use, on becoming ‘intelligent viewers’ and being advocates for reducing media use. Parents received newsletters on strategies to reduce TV/video and video game use. *Parental skills*: Parents were encouraged to motivate their children to stay within their budget. *Goal setting*: Children were encouraged by the research staff to limit their TV viewing to 7 hours per week.	School; family/home	6 months	No	Assessment only
Todd et al. [41]	Intervention *n* = 11; 100 %M; 10.0 ± 0.8 yrs Control *n* = 10; 100 %M; 9.7 ± 1.2 yrs	*General description:* Children engaged in a seminar to enhance awareness of electronic media use and to set goals to minimize use. Awareness and strategies to help minimize media use included: a 90-minute family-centred interactive session, three follow-up newsletters, TV allowance, ENUFF software to limit computer and internet use, follow-up phone call to ensure installation of TV Allowance and ENUFF software, recommendation for progressive reduction in electronic media use to 90 min per day or less in the first 10 weeks. Parents were contacted weekly by phone calls to encourage and reinforce compliance with the intervention strategy. *TV control device:* Families received a TV allowance (up to 2 per family) *Knowledge transfer:* Families engaged in interactive session and parents received three newsletters. *Goal setting:* Children set goals to minimize use (seminar) and were recommended by the research staff to limit media use to 90 minutes per day or less in the first 10 weeks.	Family/home	20 weeks	No	Only data collection
Verloigne et al. [14]	Overall 10.9 ± 0.7 yrs Intervention *n* = 141; 40 %M Control *n* = 231; 39 %M Children from Belgium	*General description:* Children engaged in one or two lessons per week (at school) on reducing screen time and breaking up sitting time (UP4FUN) covering one specific theme each week: 1) Introduction of the project, 2) awareness of sitting time, 3) evaluation of sitting time, 4) influencing factors at home, 5) possibilities for activity breaks and active transportation, 6) Family Fun Event. To increase parental involvement, the teacher handed out a weekly newsletter to the children containing personalized messages of the children and homework tasks. Motivational factors included the ‘fun’ aspect of the intervention (e.g. step counters and stickers) and public commitment to the project message (by UP4FUN bracelets). *Theory-based*: Social ecological perspective. *Knowledge transfer:* Children engaged in lessons on SB. Parents received six weekly newsletters. *Child involvement:* Activities such as making list of non-sedentary activities, writing personal goals, discussing family screen time rules. *Goal setting*: Based on sitting time in week 2, children set personal goals.	School	6 weeks	No	Usual curriculum
Vik et al. [48]	Intervention: *n* = 1569 Control: *n* = 1578 Overall: 49 %M; 11.2 yrs From 5 European countries	UP4FUN intervention, for intervention characteristics see description given above (Verloigne et al.).				

Abbreviations: *CI* confidence interval, *h/d* hours per day, *h/wk* hours per week, *min/d* minutes per day, *M* males, *PC* personal computer, *SB* sedentary behaviour, *TV* television

^a^ Indicates the sedentary behaviour group

^b^ Indicates the intervention additionally targeted a healthy diet [13, 32, 37, 38] and/or adequate sleep [13]

**Table 2:** Sedentary behaviour outcome measures and results of intervention targeting exclusively sedentary behaviour – sorted by age range and methodological quality

Ref	Quality rating^a^	SB outcome	Results ^§^
Children aged 2.5 – 7 years			
Birken et al. [11]	Moderate	Parent-reported total time (min/d) the child was in a room with the TV/video/DVD on or playing video games or using the Internet during previous weekday and weekend day.	Adjusted (baseline SB values and zBMI (WHO)) mean differences [95 % CI]: Weekday screen time (min/d): -7 [-38; 23] Weekend day screen time (min/d): 2 [-16; 20]
Dennison et al. [32]	Moderate	Parent-reported average amount of time (h/wk) watching TV/videos, playing video or computer games, or surfing the Internet, separately for Saturday, Sunday and an average weekday.	Adjusted (age, sex, baseline SB values) difference in mean change [95 % CI]: TV/video viewing (h/d): Weekdays: -0.62 [-1.11; -0.12] Saturday: -0.63 [-1.44; 0.17] Sunday: -0.99 [-1.73; -0.25] Percentage children watching >2 h/d: -21.5 [-42.5; -0.5] Computer/video game playing (h/d): Weekdays: -0.11 [-0.34; 0.13] Saturday: -0.07 [-0.49; 0.34] Sunday: -0.03 [-0.27; 0.21]
Epstein et al. [33]	Moderate	Objectively assessed (TV allowance) TV and computer time (h/wk).	Decrease in mean (SEM [SD]) number of hours of TV viewing and computer games (h/wk): Intervention: 17.5 [7.0] at 6 months and about the same through 24 months Control group: -5.2 [11.1] at 24 months Significant different changes from baseline between groups at 6 through 24 months (adjusted for group, SES, age, sex).
Haines et al. [13]	Weak	Parent-reported time their child watched TV on average weekday and weekend day in the past month (h), and whether child had a TV in the bedroom.	Mean group difference [95 % CI] for changes from baseline to 6 months: TV time (h/d): 0.54 [-1.22; 0.15] TV time on weekdays (h/d): -0.31 [-0.98; 0.37] TV time on weekend days (h/d): -1.06 [-1.97; -0.15] Number of TVs in bedroom (OR): 1.75 [0.62; 4.91]
Taveras et al. [34]	Weak	Child-reported TV and video viewing (h/d), TV in bedroom (y/n).	Adjusted (age, sex, ethnicity, parent education, overweight/obesity status at baseline, household income, time elapsed from baseline to follow-up) difference (b [95 % CI]): Total TV/video viewing (h/d): -0.36 [-0.64; 0.09] Odds ratio (OR [95 % CI]): TV in bedroom (%): 0.65 [0.32; 1.32] (additionally adjusted for TV in bedroom at baseline)
Yilmaz [36]	Weak	Parent-reported (h/wk) time spent watching TV, videos or surfing internet. Parent reported (h/d) time spent in front of a screen, for weekend and weekdays separately.	Media time at 2, 6 and 9 months significantly different between intervention and control group.
Zimmerman et al. [35]	Weak	Parent-reported time diaries (15-minute segment for the entire 24-h day) for one randomly chosen weekday and one randomly chosen weekend including their child’s total TV viewing time (min/day) and commercial TV viewing time (min/day) (by indicating name of the show and media format (i.e. TV/DVD)).	Beta [95 % CI] for intervention effect: Total TV viewing time (min/d): -37.1 [-68.7; -5.6] Commercial TV viewing (min/d): -29.2 [-63.0; 4.6]
Children aged 7 – 12 years			
Ford et al. [40]	Moderate	Parent-reported the child’s typical weekday and Saturday TV/video and video game use (h), nr of days the child had breakfast/dinner while watching TV and overall household TV use (h).	Effects sizes (Cohen’s δ^§^) for baseline to post-test differences (all non-significant): Mean weekly screen use (h): 0.00 Overall household TV use: 0.20 Days breakfast with TV on: 0.26 Days dinner with TV on: 0.45
Hinckson et al. [46]	Moderate	Objectively measured (ActivPAL) time spent sitting and sit-to-stand counts.	Mean group difference (intervention minus control) for changes from pre to post intervention [90 % confidence limits]: Sitting (h): -0.49 [0.64] Sit-to-stand counts: -0.96 [0.54]
Maddison et al. [47]	Moderate	Child-reported time spent (min/d) sedentary, screen-based and non screen-based (Multimedia Activity Recall for Children and Adolescents, MARCA).	Mean difference (intervention minus control) for changes from pre to post intervention [95 % CI]: Total SB (min/d): -20 [-56; 17] Screen-based SB (min/d): -33 [-73; 7] Non screen-based SB (min/d): 13 [-26; 51]
Robinson et al. [43]	Moderate	Child- and parent-reported (h/wk) TV/video viewing and video game playing, number of meals and snacking with TV ON, and time spent (h/d) in other SB (i.e. using a computer, doing homework, reading, listening to music, playing a musical instrument, talking with parents, playing quiet games indoors and at classes or clubs. Parent reported overall household TV use.	Adjusted (baseline SB, age, sex) change [95 % CI]: Child report TV (h/wk): -5.53 [-8.64; -2.42] Videotapes (h/wk): -1.53 [-3.39; 0.33] Video games (h/wk): -2.54 [-4.48; -0.60] Meals while TV ON (nr): -0.54 [-0.98; -0.12] Snacking while TV ON (nr): -0.11 [-0.27; 0.04] Other SB (h/d): -0.34 [-1.21; 0.52] Parental report TV (h/wk): -4.29 [-5.89; -2.70] Videotapes (h/wk): -0.25 [-1.19; 0.69] Video games (h/wk): -0.76 [-1.75; 0.22] Meals while TV ON (nr): -1.07 [-1.69; -0.18] Children snacking while TV ON (%): -1.94 [-9.06; 5.17] Other SB (h/d): -4.88 [-11.69; 1.93] Overall household TV use: -0.77 [-1.69; 0.14]
Vik et al. [48]	Moderate/Weak^b^	Objectively measured (Actigraph GT1M, GT3X or ActiTrainer) breaks in SB and total SB. Self-reported breaks in sitting time and screen time spent, separate for TV/DVD hours, PC/games console hours and school hours.	Adjusted (school, age, baseline SB) means [95 % CI]: Breaks in SB (nr/day): Objective: 0.17 [-1.18; 1.52] Self-reported TV/DVD: 0.14 [0.02; 0.25] Self-reported PC/games: 0.13 [0.02; 0.24] Self-reported school hours: 0.10 [-0.04; 0.23] Total SB (h/d): Objective: 0.11 [-0.11; 0.33] Self-reported FQ TV/DVD: -0.03 [-0.12; 0.05] PC/games: -0.01 [-0.10; 0.09] Self-reported 24 h recall TV/DVD: -0.06 [-0.15; 0.03] PC/games: 0.02 [-0.08; 0.12]
Cardon et al. [44]	Weak	Observations on durations and frequencies of static and dynamic sitting (portable ergonomic observation method).	Mean [SD] frequencies and durations (%) post intervention (except for frequency static sitting all outcomes significant different between intervention and control group): Intervention: Frequency static sitting: 1.50 [1.00] Duration static sitting: 1.0 [0.00] Frequency dynamic sitting: 13.72 [7.65] Duration dynamic sitting: 53.11 [23.23] Control: Frequency static sitting: 4.17 [4.35] Duration static sitting: 97.13 [3.82] Frequency dynamic sitting: 2.38 [2.10] Duration dynamic sitting: 3.25 [2.87]
Carson et al. [12]	Weak	Objectively measured (ActiGraph GT3X) classroom and total sedentary time (min/d).	Adjusted (sex, country of birth, SES, baseline and 24-month accelerometer wear time, baseline mediator variables) beta [95 % CI]: Classroom sedentary time (min/d): -0.17 [-6.14; 6.48] Total sedentary time (min/d): -6.9 [-19.50; 5.69]
Epstein et al. [38]	Weak	Self-reported physical activity questionnaire (Minnesota Leisure Time Activity Survey) assessing frequency and average time spent on targeted (watching TV/video, playing computer games, talking on the phone, playing board games) and non-targeted (homework, schoolwork) SB.	Changes from baseline to 6 and 24 months (mean (SD)): Targeted SB (% time) 0–6 months: Low dose SB: -15.1 (19.0) High dose SB: -20.3 (29.4) 0–24 months: Low dose SB: -0.6 (25.2) High dose SB: -12.0 (24.7) No significant differences across groups: 0–6 months: -13.4 (22.6) 0–24 months: -8.7 (23.6) Non-targeted SB (% time) 0–6 months: Low dose SB: 11.1 (24.7) High dose SB: 10.5 (17.8) 0–24 months: Low dose SB: -2.1 (23.4) High dose SB: 2.4 (16.6) No significant differences across groups: 0–6 months: 9.3 (18.7) 0–24 months: 1.2 (20.2)
Epstein et al. [37]	Weak	Child- and parent-report of any SB that took 10 min or longer in duration, using index cards (structured with columns for start and stop times and the activity description)	Sign decrease in SB over time (-2.2 ± 7.4; % time in targeted sedentary behaviours), with no differences between groups.
Escobar-Chaves et al. [39]	Weak	Parent-reported media use (h, min) by children (TV/DVD, video/computer game, computer use, handheld games), media in household and in child’s bedroom, frequency of TV ON when nobody was watching, frequency of TV on while eating snacks/meals.	Adjusted (gender, age and ethnicity) OR: Media use (i.e. TV/DVD/video viewing, computer game/use): non-significant difference TV being ON when nobody was watching: 0.23 (significant) Eating snacks while watching TV: 0.47 (significant) TV in the child's bedroom: 0.23 (significant)
French et al. [45]	Weak	Objectively assessed TV viewing time (h/d; TV control device) and sedentary time (Actigraph GT1M).	Mean values [SE] post intervention (significantly different between intervention and control for TV viewing time): Intervention: TV viewing time (h/d): 1.7 (0.2) Sedentary time (min/d): 821.0 (34.9) Control: TV viewing time (h/d): 2.6 (0.3) Sedentary time (min/d): 792.3 (43.5)
Ni Mhurchu et al. [42]	Weak	Child-reported hours of TV watching and total screen time per week (h/wk).	Mean change (mean (SD)) from baseline to 6 weeks (all non-significant): Intervention: Total weekly TV viewing (h): -254 (536) Total weekly screen time (h): -706 (725) Control: Total weekly TV viewing (h): -254 (536) Total weekly screen time (h): -706 (725)
Todd et al. [41]	Weak	Recalled (by participant) all non-school related electronic media use (including that at friends’ homes and elsewhere), both time (h/min) and type (e.g. TV, computer).	Adjusted (media access, participation in organized activities) difference [95 % CI]: Electronic media use (min/d): Baseline to 10 weeks: -83 [-92.2; -73] Baseline to 20 weeks: -73 [-78.5; -67.5] Significant treatment x time interaction Nr of meals and snacks per day with electronic media ON decreased to 70 % below baseline at 10 and 20 weeks in intervention group compared to 10 % decrease at 10 weeks and 40 % increase 20 weeks in control group (significant different between groups).
Verloigne et al. [14]	Weak	Accelerometer measured sedentary time (% wearing time), worn for at least 2 weekdays (10 h wearing time) and 1 weekend day (8 h wearing time).	Adjusted (age, gender) b (SE) for interaction between ‘time’ and ‘condition’ for sedentary time outcomes (% wearing time) (all non-significant): Day: 0.96 (0.86) Weekday: 1.48 (0.78) Weekend day: 0.03 (1.41) School hours: 0.70 (0.75) After school hours: 1.69 (1.09) Sedentary bouts: -0.28 (0.23)

^§^Cohen’s δ: standardized effect size = mean change scores for two groups/pooled within-group standard deviation

Abbreviations: *BMI* body mass index, *CI* confidence interval, *h/d* hours per day, *h/wk* hours per week, *min/d* minutes per day, *OR* odds ratio, *PC* personal computer, *SB* sedentary behaviour, *SD* standard deviation, *SE* standard error, *SEM* standard error of the mean, *SES* socioeconomic status, *TV* television

^a^Quality assessment tool for quantitative studies, Effective Public Health Practice Project (EPHPP)

^b^M for accelerometer assessed outcomes, W for self-reported outcomes

In eight studies [33, 34, 40–43, 45, 47] a TV/computer control device was used to budget time spent on TV/DVD viewing, computer use and playing computer games. In three studies a TV turnoff period (ranging from 7 to 10 days) was encouraged [11, 32, 43]. Ten interventions were in the family/home setting [11, 13, 35, 36, 39–42, 45, 47] and seven interventions in the school/pre-school/day-care setting [12, 14, 32, 43, 44, 46], of which three interventions additionally involved the family/home setting [12, 32, 43]. Four studies were in the clinical setting, involving the family/home setting as well [33, 34, 37, 38]. Ten interventions were theory based [11, 12, 14, 34–36, 40, 43, 47, 48], of which six studies were based on the Social Cognitive Theory [12, 35, 36, 40, 43, 47]. All but two [45, 46] included studies included knowledge transfer as intervention strategy, of which 11 studies targeted parents [11, 13, 32, 33, 35, 36, 39, 40, 42, 47, 48], one study targeted the children [44] and eight studies both the children and their parents [12, 14, 34, 37, 38, 41, 43, 48]. Parental skills were targeted in 14 studies [11, 13, 32, 33, 35–40, 42, 43, 45, 47], including monitoring, goal setting and rewarding their child’s behaviour. Twelve studies applied goal setting within the intervention, with goals set by the research staff or parents (five studies [13, 33, 35, 42, 45]), by the children themselves (five studies [14, 37, 41, 43, 48]) or both by researchers/parents and children [38]. Three studies introduced furniture that encouraged children to sit less and move more (e.g. standing desks, mats) [12, 44, 46].

Table 2 shows the sedentary behaviour outcome measures of all included studies. Most (15 out of 21) studies used a parent- or child-reported measure of sedentary time. Of these studies, three studies were limited to TV/DVD time [13, 34, 35], seven studies assessed screen time [11, 32, 36, 39–42] and four studies included a broad range of sedentary behaviours, including reading, doing homework, artwork, crafts or being sedentary at school [37, 38, 43, 48]. Four studies used accelerometers [12, 14, 45, 48] and one study used the ActivPAL [46] to assess sedentary and sitting time, respectively. Two studies used a TV/computer allowance [33, 45] to assess both TV and computer time, and one study used the Portable Ergonomic Observation (PEO) method [44]. A number of trials also provided some information or counselling on sedentary behaviour in the control group; as a result there may have been insufficient contrast between the intervention and the control group.

#### Methodological quality and intervention effects

Additional file 3 shows the quality assessment scores and Table 2 shows the results of all included studies (sorted by age range and methodological quality). None of the studies were rated as being of strong methodological quality, six studies were moderate and 12 studies were of weak quality. Except for one study [44], all studies were randomized controlled trials, indicating adequate study designs. In contrast, study samples were often not representative, assessors not blinded to group assignment, participants not blinded to the research question(s) and the outcome measures were of unknown/inadequate validity and reliability. Moreover, participation rate, intended intervention dose, analyses according to intention-to-treat and method of randomization were poorly described.

Of the eight moderate quality studies, four studies reported significant intervention effects on children’s sedentary time [32, 33, 43, 46] while four studies reported no significant effects [11, 40, 47, 48]. Among children aged 2.5 to 5.5 years old, Dennison et al. [32] applied a 7-week intervention targeting children’s TV viewing. Intervention strategies included knowledge transfer (take-home messages of sessions and brochures), a TV turnoff period of one week and targeting parental skills (monitoring and rewarding their child’s behaviour). This study found that significantly fewer children in the intervention group watched >2 h per day TV than control children (parent-report). Moreover, they found that children in the intervention group watched significantly less TV/video on weekdays and Sundays (i.e. -36 and -60 min/day, respectively) than control children, but not on Saturdays. No significant intervention effects were found on computer/video game use in this study [32]. Epstein et al. [33] found that, after a 2-year intervention period targeting both TV and computer time, 4-to-7 year old children (BMI >75^th^ percentile for age and sex) in the intervention group spent less time on objectively assessed (i.e. TV/computer allowance) TV viewing and computer games (i.e. -3.2 h/d) than children in the control group. This study included knowledge transfer (monthly newsletters to parents), the use of a TV control device, parental skills (rewarding their child’s behaviour) and goal setting (goals set by research staff), as intervention strategies. Among 9-to-10 year olds, Hinckson et al. [46] implemented standing desks in classrooms of the intervention school for four weeks. Compared to children in the control classroom, children in the intervention classroom reduced their overall time spent sitting though the difference was small [46]. Among 8-to-9 year olds, Robinson et al. [43] applied a 6-month intervention targeting decreasing media use. Intervention strategies included a 10-day TV turnoff period, the use of a TV control device, knowledge transfer (children engaged in lessons, parents received newsletters), parental skills (rewarding their child’s behaviour) and goal setting (goals set by research staff). This study found that children in the intervention group reported watching less TV (i.e. 47 min/d) and playing fewer videogames (i.e. 22 min/d) than control children, however, no effects were found on watching videotapes and other sedentary behaviours (i.e. using a computer, reading, listening to music). Based on parent-report, only children’s TV viewing was significantly lower in the intervention group than the control group (i.e. -37 min/d) [43].

According to the best-evidence synthesis, based on the eight moderate quality studies, we found conflicting/inconsistent evidence for the effectiveness of interventions targeting sedentary behaviour. Nevertheless, from these moderate quality studies we identified two promising intervention strategies (i.e. moderate evidence). Second, one study in which standing desks were implemented in classrooms demonstrated promising intervention effects [46].

### Discussion

This review aimed to summarize the evidence on the effectiveness of interventions targeting exclusively sedentary behaviour in children aged 0–18 years. We conclude that there is conflicting/insufficient evidence for the effectiveness of such interventions. Based on the three moderate quality studies reporting significant intervention effects, two intervention strategies seem promising: 1) encouragement of a TV turnoff week; and 2) implementation of standing desks in classrooms. Since there were only eight moderate quality studies, including children with a wide age range (i.e. varying from 3.1 to 11.2 years), we could not draw age-specific conclusions.

Two out of three studies that included the encouragement of a TV turnoff week as a strategy to reduce sedentary time found reductions on TV/video time [32, 43]. Encouraging children not to watch TV for a certain time period may help them to experience the enjoyment of behaviours other than TV viewing. A premise for this is that the alternative behaviour should be as fun as the specific sedentary behaviour [49]. Robinson et al. [43] found no effects on other sedentary behaviours such as watching videotapes, reading and using the computer, indicating that TV viewing was not replaced by other sedentary activities. Future studies should confirm the potential of implementing a TV turnoff week to reduce TV viewing time and explore longer-term effects.

Recently, the implementation of furniture nudging interruptions in sedentary time, for example standing breaks, within the school setting has gained more attention. Although the effect was small, Hinckson et al. [46] found a reduction in overall time spent sitting when implementing standing desks. To date, it is unclear whether standing interruptions can prevent potential negative health effects of excessive sedentary behaviour in children.

Surprisingly, although in a number of studies children were involved in implementing the intervention (e.g. setting personal goals, discussing non-sedentary activities), no studies collaborated with children and/or parents in the development of interventions. It is likely that active participation of both children and their parents in the choice and development of intervention strategies may lead to more acceptable and attractive strategies and thereby more effective interventions [50].

We aimed to identify effective intervention strategies to reduce sedentary behaviour. Unfortunately, strategies were not always clearly described. Besides being able to reliably extract intervention strategies for systematic reviews, clear descriptions of behaviour change techniques may also benefit accurate replication, faithful implementation and well-designed development of interventions [51]. We therefore recommend future studies to make use of clearly described standardized behaviour change techniques when designing and reporting intervention content.

Only four out of eight moderate quality studies reported significant intervention effects. This is in contrast with previous reviews and meta-analyses on interventions targeting sedentary behaviour in combination with physical activity promotion, in which predominantly small but significant effects and effect sizes were reported [15, 17, 18, 21–23, 25]. One explanation may be that a combined focus on reducing sedentary behaviour and increasing physical activity is more effective. Secondly, in contrast to previously published reviews, we performed a methodological quality assessment and excluded the studies with weak quality from our evidence synthesis, of which a number of studies reported some significant findings.

Only one previous review additionally summarized interventions targeting solely sedentary behaviour [19]. In contrast to our findings, Leung et al. [19] concluded that sedentary behaviour interventions significantly reduced sedentary time. Their review was based on only three studies, whereas we included 21 studies in our review. Additionally, Leung et al. [19] did not include the methodological quality of the studies in their evidence synthesis.

We found no studies of strong methodological quality. Representativeness of the study sample, controlling for relevant confounders, blinding and measurement of sedentary behaviour were issues that limited the quality of the included studies. We recommend that future studies keep these potential sources of bias in mind when designing a trial. For example, analyses should preferably adjust for baseline levels of sedentary behaviour. Regarding blinding, assessors should be blinded to group assignment, and participants should preferably be blinded to the research question or the authors could speculate on the effect of any suspected modifying factors, such as belief in the intervention, in the discussion. Finally, sedentary behaviour should be assessed through accurate, valid, reliable and responsive measures.

Strengths of this systematic review include the focus on interventions exclusively targeting sedentary behaviour. This is important when examining the true effectiveness of sedentary behaviour interventions and optimizing future interventions. Another strength of this review is that we not only assessed the methodological quality of included papers, but also took this into account in our evidence synthesis. A limitation of every review is that our findings may suffer from publication bias. Additionally, we did not calculate effect sizes as the number of included studies with a moderate or high quality was limited (i.e. eight studies) and these studies were rather heterogeneous, i.e. intervention durations ranged from 4 weeks to 2 years, age of included children ranged from 3.1 to 11.2 years and outcome measure of sedentary time reflected parent-reported total screen time, TV time, computer time, parent- and child-reported total sedentary time and objectively measured sedentary time (ActivPAL, Actigraph). Finally, due to the wide age range of included samples in the moderate quality studies, we could not draw age-specific conclusions.

## Conclusions

We conclude that to date there is unconvincing evidence for the effectiveness of interventions targeting exclusively sedentary behaviour. Based on the eight moderate quality studies that found a significant intervention effect, encouraging a TV turnoff week and implementing standing desks in classrooms seem promising. As all included studies applied multiple intervention strategies, it is impossible to distinguish which strategies are most promising. We recommend that future studies explore which strategies are most effective, by applying mediation analyses. Moreover, in order to increase the effectiveness of interventions, knowledge of children’s motives to engage in sedentary behaviour is required as well as their opinion on potentially effective intervention strategies.

## Abbreviations

%M, percentage males; b, beta; BMI, body mass index; CI, confidence interval; DVD, digital videodisc; EPHPP, Effective Public Health Practice Project; h/d, hours per day; h/wk, hours per week; min/d, minutes per day; OR, odds ratio; PA, physical activity; PC, personal computer; SB, sedentary behaviour; SD, standard deviation; SE, standard error; SEM, standard error of the mean; SES, socioeconomic status; TV, television; WHO, World Health Organization; yrs, years

## Additional files

Additional file 1: Full search for systematic review on the effectiveness of interventions targeting solely sedentary behavior. (DOCX 14 kb) Additional file 2: Methodological quality assessment tool [38]. (DOCX 18 kb) Additional file 3: Methodological quality assessment of included studies. (DOCX 57 kb)
