# Supplementary material for: Effectiveness of intervention strategies exclusively targeting reductions in children’s sedentary time: a systematic review of the literature
Source: Int J Behav Nutr Phys Act. 2016 Jun 9;13:65. doi: 10.1186/s12966-016-0387-5 (PMC4899905; doi:10.1186/s12966-016-0387-5)
Supplement: Additional file 1: — Full search for systematic review on the effectiveness of interventions targeting solely sedentary behavior. (DOCX 14 kb) [file 12966_2016_387_MOESM1_ESM.docx]

**Additional file 1.** Full search for systematic review on the effectiveness of interventions targeting solely sedentary behavior.

**Search in Pubmed**

(Intervention OR Randomized Controlled Trial OR control group OR controlled trial) AND

(sedentary behaviour OR sedentary behaviours OR sedentary behavior OR sedentary behaviors OR sedentary time OR sedentary lifestyle OR sitting time OR prolonged sitting OR computer time OR computer use OR screen time OR screen-time OR sedentary activity OR sedentary activities OR television OR gaming) AND

(child OR adolescent OR preschool child OR youth OR schoolchild OR youths OR pediatri* OR paediatr* OR boy OR boys OR girl OR girls OR teen OR teens OR teenager* OR puberty OR infant OR baby OR babies OR toddler* OR preschool*)

**Search in Cochrane**

(Intervention OR Randomized Controlled Trial OR control group OR controlled trial) AND

(sedentary behaviour OR sedentary behaviours OR sedentary behavior OR sedentary behaviors OR sedentary time OR sedentary lifestyle OR sitting time OR prolonged sitting OR computer time OR screen time OR screen-time OR sedentary activity OR sedentary activities OR television OR gaming) AND

(child OR adolescent OR preschool child OR youth OR schoolchild OR youths OR pediatri* OR paediatr* OR boy OR boys OR girl OR girls OR teen OR teens OR teenager* OR puberty OR infant OR baby OR babies OR toddler* OR preschool*)

**Search in EMBASE**

(intervention OR randomized AND controlled AND trial OR 'control'/exp OR control AND group OR controlled) AND

(sedentary AND ('behaviour'/exp OR behaviour) OR sedentary AND behaviours OR sedentary AND ('behavior'/exp OR behavior) OR sedentary AND behaviors OR sedentary AND ('time'/exp OR time) OR sedentary AND ('lifestyle'/exp OR lifestyle) OR 'sitting'/exp OR sitting AND ('time'/exp OR time) OR prolonged AND ('sitting'/exp OR sitting) OR 'computer'/exp OR computer AND ('time'/exp OR time) OR screen AND ('time'/exp OR time) OR 'screen time' OR sedentary AND activity OR sedentary AND activities OR 'television'/exp OR television OR gaming)

AND ('child'/exp OR child OR 'adolescent'/exp OR adolescent OR preschool AND ('child'/exp OR child) OR 'youth'/exp OR youth OR 'schoolchild'/exp OR schoolchild OR youths OR pediatri* OR paediatr* OR 'boy'/exp OR boy OR boys OR 'girl'/exp OR girl OR girls OR teen OR teens OR teenager* OR 'puberty'/exp OR puberty OR 'infant'/exp OR infant OR 'baby'/exp OR baby OR babies OR toddler* OR preschool*)
