# Supplementary material for: Effectiveness of intervention strategies exclusively targeting reductions in children’s sedentary time: a systematic review of the literature
Source: Int J Behav Nutr Phys Act. 2016 Jun 9;13:65. doi: 10.1186/s12966-016-0387-5 (PMC4899905; doi:10.1186/s12966-016-0387-5)
Supplement: Additional file 2: — Methodological quality assessment tool [38]. (DOCX 18 kb) [file 12966_2016_387_MOESM2_ESM.docx]

**Additional file 2.** Methodological quality assessment tool[38].

1. Selection bias
   - 1. Representative sample, i.e. random selection from target population
     2. Participation rate (%) before assignment to intervention or control group
2. Study design
   1. Design of the study
   2. Randomization
   3. Method of randomization
3. Confounding
   1. Baseline differences between intervention and control group
   2. Adjusted for relevant confounders (i.e. baseline SB, physical activity, age, sex, ethnicity) when different between groups at baseline
4. Blinding
   1. Assessors blinded
   2. Participants blinded to the research question
5. Data collection methods
   1. Valid
   2. Reliable
6. Withdrawals and drop-outs
   1. Number and reasons reported
   2. Completion (%)
7. Intervention integrity
   1. Number of participants receiving the intended intervention
   2. Consistency measure, indicating whether the intervention was provided to all participants in the same way
8. Analyses
   1. Similar allocation and analysis unit
   2. Intention to treat
   3. Adequate sample size for number of variables in analysis
   4. Point estimate provided

For each subscale a rating (strong, moderate, weak) was provided, which informed the global rating.

*Global rating*

Strong: Maximum one weak scores and maximum 2 moderate scores

Moderate: Maximum two weak scores

Weak: More than two weak score
