# Supplementary material for: Effectiveness of intervention strategies exclusively targeting reductions in children’s sedentary time: a systematic review of the literature
Source: Int J Behav Nutr Phys Act. 2016 Jun 9;13:65. doi: 10.1186/s12966-016-0387-5 (PMC4899905; doi:10.1186/s12966-016-0387-5)
Supplement: Additional file 3: — Methodological quality assessment of included studies. (DOCX 57 kb) [file 12966_2016_387_MOESM3_ESM.docx]

**Additional file 3.** Methodological quality assessment of included studies.

|  | **Selection bias** | **Study design** | **Confounders** | **Blinding** | **Data collection methods** | **Withdrawals and drop outs** | **Intervention integrity** | **Analyses** | **Global rating^1^** |
| --- | --- | --- | --- | --- | --- | --- | --- | --- | --- |
| Birken et al[11] | M | S | M | S | W | M | W | S | **M** |
| Cardon et al[44] | M | S | W | W | M | S | S | W | **W** |
| Carson et al[12] | W | S | M | W | S | M | W | M | **W** |
| Dennison et al[32] | M | S | S | W | W | M | W | S | **W** |
| Epstein et al[38] | W | S | S | W | W | M | W | M | **W** |
| Epstein et al[37] | W | S | S | W | W | M | M | M | **W** |
| Epstein et al[33] | W | S | S | M | S | S | S | M | **M** |
| Escobar-Chaves et al[39] | W | S | S | W | W | M | M | M | **W** |
| Ford et al[40] | M | S | S | W | S | S | M | M | **M** |
| French et al[45] | W | S | M | W | M | M | M | W | **W** |
| Haines et al[13] | W | S | S | W | W | M | W | S | **W** |
| Hinckson et al[46] | M | S | W | M | S | M | S | M | **M** |
| Maddison et al[47] | W | S | S | W | M | M | W | S | **M** |
| Ni Mhurchu et al[42] | W | S | M | W | W | M | M | M | **W** |
| Robinson et al[43] | M | S | M | S | W | M | W | S | **M** |
| Taveras et al[34] | W | S | W | W | W | M | W | S | **W** |
| Todd et al[41] | W | S | W | M | W | S | M | S | **W** |
| Verloigne et al[14] | M | S | W | M | S | M | W | W | **W** |
| Vik et al[48] | M | S | M | W | S/W^2^ | S | W | S | **M/W**^3^ |
| Yilmaz et al [36] | W | S | W | S | W | M | W | M | **W** |
| Zimmerman et al[35] | W | S | W | W | M | W | M | M | **W** |

S, strong; M, moderate; W, weak

^1^Strong: at most 1 weak and 2 moderate scores; moderate: at most 2 weak scores; weak: more than 2 weak scores.

^2^S for accelerometer assessed outcomes, W for self-reported outcomes

^3^M for accelerometer assessed outcomes, W for self-reported outcomes
